# Supplementary material for: Mitochondrial Transfer by Human Mesenchymal Stromal Cells Ameliorates Hepatocyte Lipid Load in a Mouse Model of NASH
Source: Biomedicines. 2020 Sep 14;8(9):350. doi: 10.3390/biomedicines8090350 (PMC7554948; doi:10.3390/biomedicines8090350)
Supplement: Supplementary file 1 [file biomedicines-08-00350-s001.zip › Supplementary Material file 4_rv1.pptx]

## Slide 1
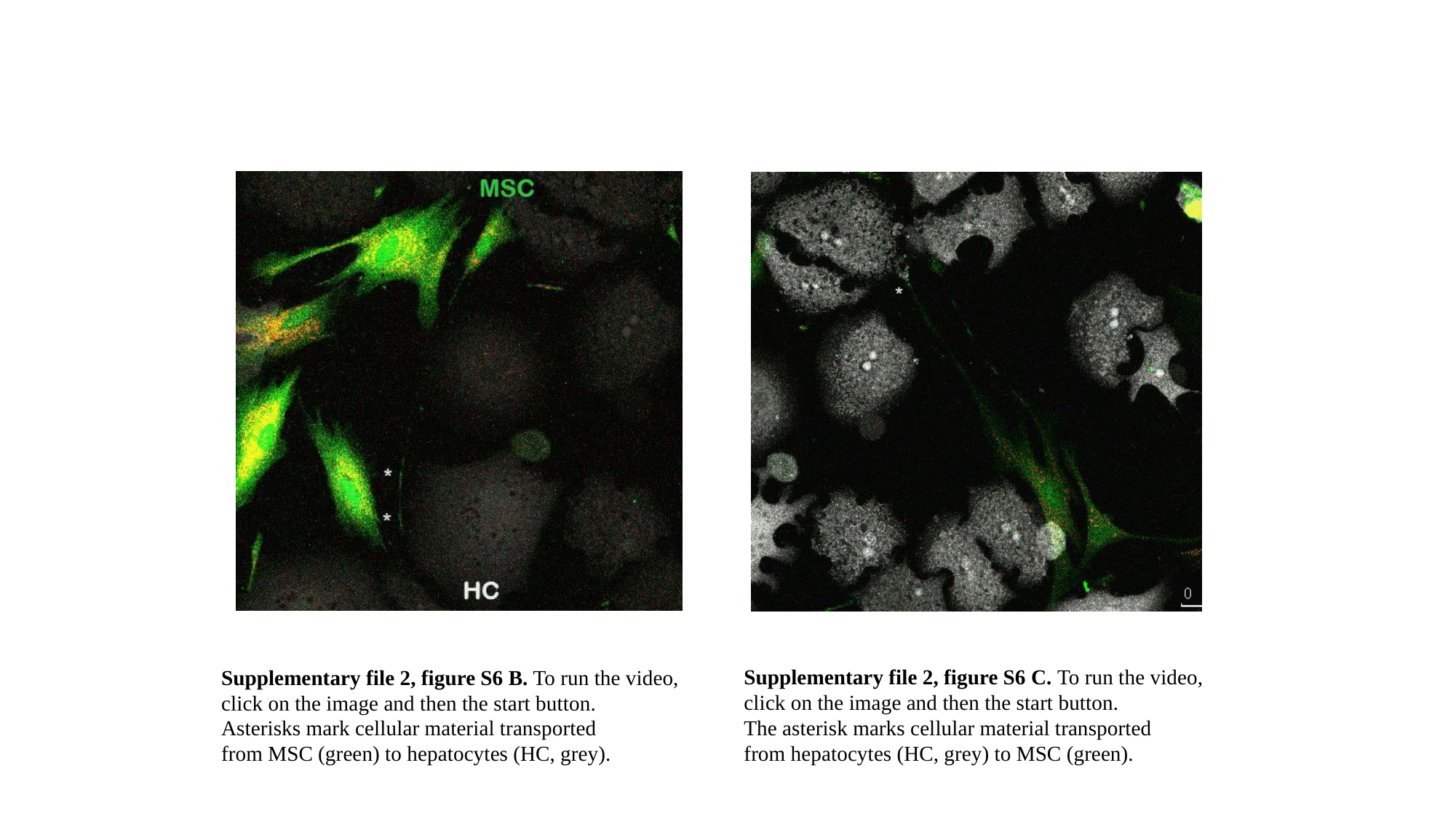

Supplementary file 2, figure S6 C. To run the video,
click on the image and then the start button.
The asterisk marks cellular material transported
from hepatocytes (HC, grey) to MSC (green).
Supplementary file 2, figure S6 B. To run the video,
click on the image and then the start button.
Asterisks mark cellular material transported
from MSC (green) to hepatocytes (HC, grey).
