# Supplementary material for: Mitochondrial Transfer by Human Mesenchymal Stromal Cells Ameliorates Hepatocyte Lipid Load in a Mouse Model of NASH
Source: Biomedicines. 2020 Sep 14;8(9):350. doi: 10.3390/biomedicines8090350 (PMC7554948; doi:10.3390/biomedicines8090350)
Supplement: Supplementary file 1 [file biomedicines-08-00350-s001.zip › Supplementary Material file 2_rv1.docx]

**Supplementary file 2**

**Table S1. List of antibodies used in this study.**

| **Antibody** | **Cat-No., Supplier** | **Dilution** | **Application** |
| --- | --- | --- | --- |
| PPARα | MA1-822, Thermo Scientific | 1:1.000 | WB |
| CD36 | Nb 400-144, Novus Bio | 1:1.000 | WB |
| Vinculin | 05-386, Merck | 1:3.000 | WB |
| Anti-rabbit-HRP | 554021, BD Biosciences | 1:7.500 | WB |
| Anti-mouse-HRP | 554002, BD Biosciences | 1:7.500  1:500 | WB  IHC |
| Cyp2e1 | ab28146, Abcam | 1:200 | IHC |
| 4-HNE | #4HNE11-S, Alpha diagnostics | 1:200 | IHC |
| Biot anti-rabbit | 111-065-003 Dianova | 1:500 | IHC |
| CD36 | Nb 400-144, Novus Bio | 1:200 | IHC |
| E-Cadherin | 610182, BD Biosciences | 1:200 | IHC |
| β-Catenin | 610154, BD Biosciences | 1:200 | IHC |
| Cyclophilin A | 2175, Cell Signaling | 1:100 | IHC |
| human mitochondria | MAB1273, Millipore | 1:400  1:200 | ICC  IHC |
| beta III Tubulin- Alexa Fluor 488 | ab195879, Abcam |  | ICC |
| Anti-mouse-488 | 115-545-003, Dianova | 1:500 | IHC |
| Anti-rabbit 488 | 4412, Cell Signaling | 1:500 | IHC |
| Anti-mouse Cy3 | 115-165-003, Dianova | 1:200 | IHC |
| Anti-rabbit-568 | A11036, Life Technologies | 1:500 | IHC |

WB, Western blot; IHC, Immunohistochemistry; ICC, immunocytochemistry

**Table S2.** **Primer pairs used for PCR.**

**Human Primers**

|  | **F** | **R** | **Tm (°C)** | **cycles** | **bp** |
| --- | --- | --- | --- | --- | --- |
| B2M | TGTCTTTCAGCAAGGACTGGT | TTCAAACCTCCATGATGCTGC | 55 | 19 | 161 |
| RHOT1 | ATCCAGAGAGGGAGACACGA | GCTCATCAGGTGACAAAGCA | 55 | 26 | 211 |
| RalA | CAGACAGCTATCGGAAGAAG | AGAAAACACAGAGGAACCCC | 55 | 25 | 138 |
| KIF5B | ACCTGCGCAAACTCTTTGTT | TCGAAGTCGCTTTTCCAACT | 55 | 25 | 212 |
| TNFaip2 | GAAGTCTGGCTGAGGTCTGG | CTCCAGAAGGAGTGCAGGAC | 57 | 31 | 201 |
| PPARα | AAAACACCAGCCTCCAGATG | GCTAACTGCAGAGGGTGAGG | 60 | 27 | 206 |
| TFAM | AGCTCAGAACCCAGATGC | CCACTCCGCCCTATAAGC | 54 | 27 | 115 |
| HMOX1 | CGGGCCAGCAACAAAGTG | AGTGTAAGGACCCATCGGAGAA | 57-60 | 27 | 107 |
| PPARGC1A | GCTACGAGGAATATCAGCACGA | ACACGGCGCTCTTCAATTG | 55.9 | 33 | 127 |

**Mouse Primers**

|  | **F** | **R** | **Tm (°C)** | **cycles** | **bp** |
| --- | --- | --- | --- | --- | --- |
| B2M | TCTACTGGGATCGAGACATGTGA | ATTGCTATTTCTTTCTGCGTGCAT | 59 | 17 | 124 |
| Acaa1a | CCCAATTTCTGAGCGGCATC | AGATATTCCCAGGGTTCCCCA | 60 | 24 | 190 |
| Acaa1b | CCGGGGCTGGAGCCATTAT | TCTGATGCCACCAGCAATGTT | 59 | 27 | 134 |
| ACLY | CCCAAGTCCAAGATCCCTGC | CTGCTTGTGATCCCCAGTGA | 59 | 24 | 181 |
| Albumin | CCAATCCTCCCGCATGCTAC | GCGAACTAGAATGGCATTTTGGAA | 59 | 16 | 140 |
| CPT1 | CCATGATGGACCCCACAACA | ATGGCTCAGACAGTACCTCCT | 59 | 24 | 172 |
| CYP2E1 | ACAAGAACAAGGGGATTATTTTCAA | AGGTAGGGTCAAAAGGCTGG | 59 | 24 | 216 |
| FABP1 | TGAAGGCAATAGGTCTGCCC | TCACCTTCCAGCTTGACGAC | 59 | 24 | 208 |
| FASN | TGCACCTCACAGGCATCAAT | GTCCCACTTGATGTGAGGGG | 59 | 24 | 104 |
| HMGCR | TTGGAGTTGGCACCATGTCA | TCACGAGAAAGCTCTAGGACC | 59 | 24 | 139 |
| HMOX1 | AGCACTATGTAAAGCGTCTC | CGGTCTTAGCCTCTTCTGT | 51.5-54.9 | 55 | 282 |
| MTTP | GAAAGAAATTGAAATGCGAAACAGA | GGTTAACACTGCAAACCAGATGAAT | 59 | 24 | 196 |
| PPARα | GTTCACGCATGTGAAGGCTG | AGCGAATTGCATTGTGTGACAT | 59 | 24 | 172 |
| PPARGC1A | ACTGAGCTACCCTTGGGATG | TAAGGATTTCGGTGGTGACA | 51-54.9 | 27 | 112 |
| SREBP1c | GATTTGGCCCGGGGAGATTT | TGGCGGATGAGGTTCCAAAG | 59 | 24 | 128 |
| SREBP2 | TCATGGGGACAGATGCCAAG | TGCCCTTCAGGAGCTTGTTTTT | 59 | 28 | 150 |
| TFAM | ATTCCGAAGTGTTTTTCCAGCA | TCTGAAAGTTTTGCATCTGGGT | 53-56.4 | 24 | 122 |


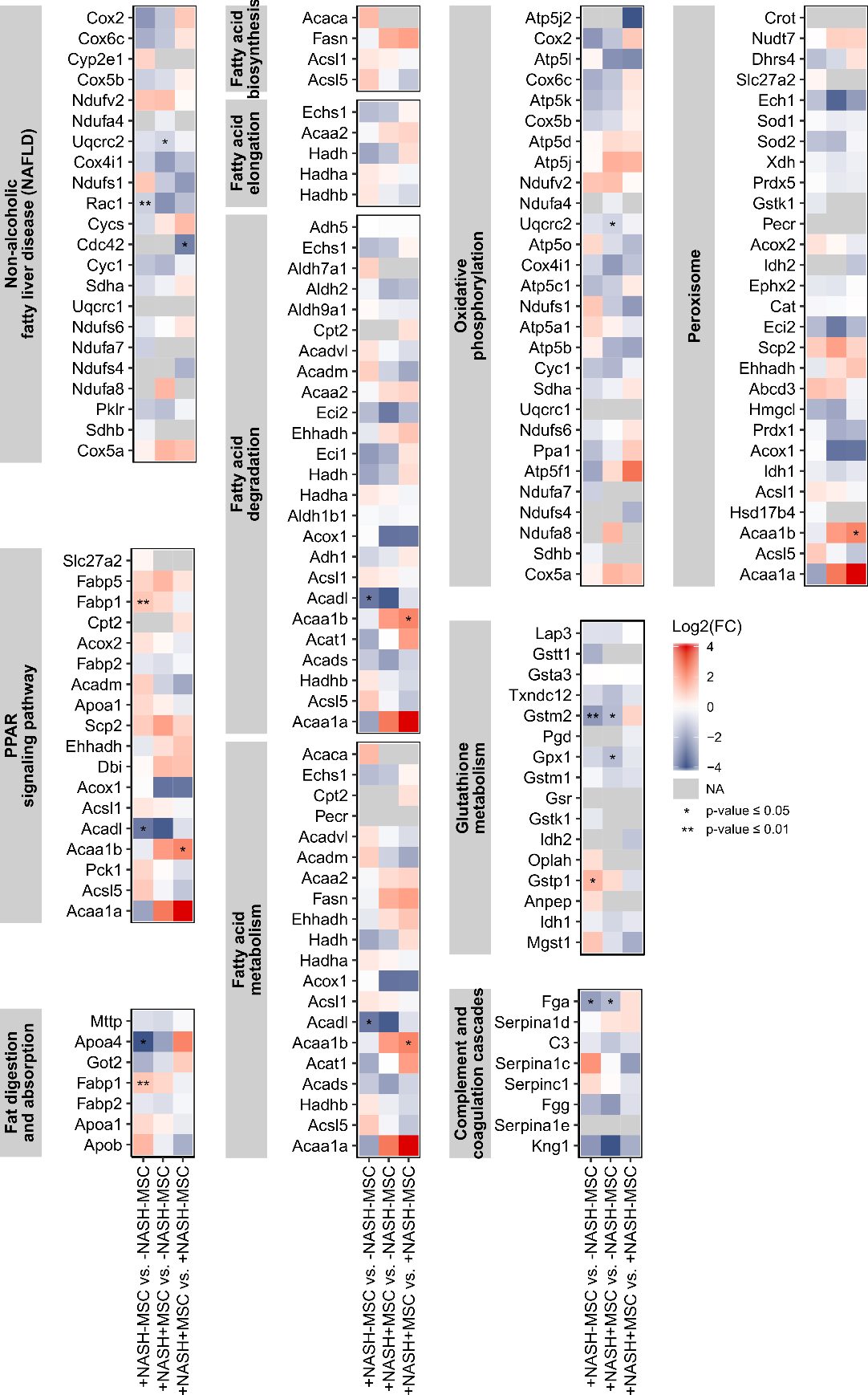
**Figure S1. Mapping of proteins to KEGG pathways.**

Shown are Log2(FCs) and p-values (p-value ≤ 0.05: *, p-value ≤ 0.01: **) of proteins that can be assigned to the following KEGG pathways: mmu03320: PPAR signaling pathway, mmu04975: Fat digestion and absorption, mmu04610: Complement and coagulation cascades, mmu00190: Oxidative phosphorylation, mmu00480: Glutathione metabolism, mmu04932: Non-alcoholic fatty liver disease (NAFLD), mmu04146: Peroxisome, mmu00061: Fatty acid biosynthesis, mmu00062: Fatty acid elongation, mmu01212: Fatty acid metabolism, and mmu00071: Fatty acid degradation.


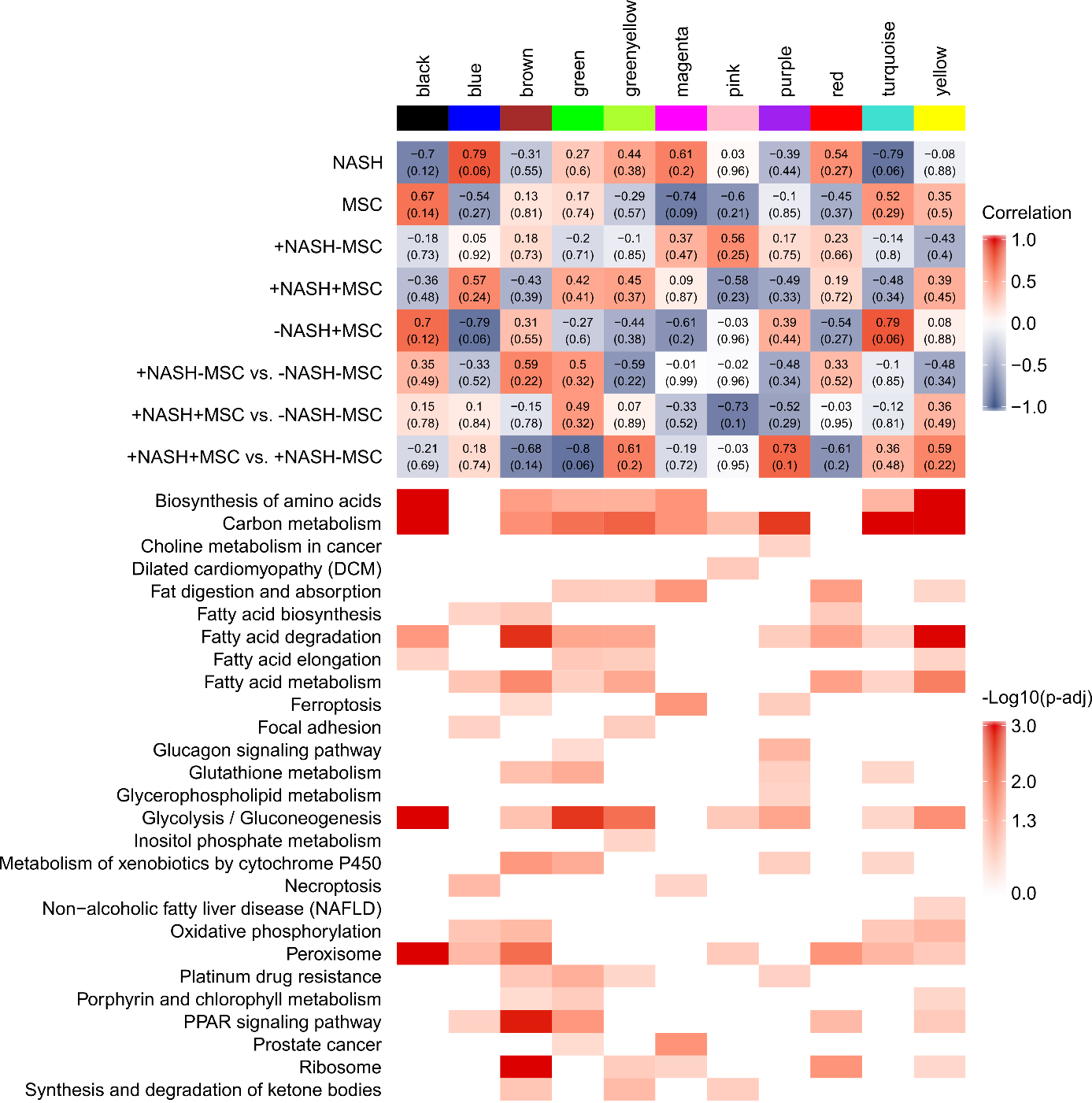
**Figure S2: Complete module-trait correlation matrix and enriched KEGG pathways.**

Shown are module-trait correlation values and *p*-values in brackets for all modules identified with WGCNA. Furthermore, the top 2 enriched pathways (based on the adjusted *p*-values) are assigned to each module. The list of the top 2 pathways was extended by pathways, of which selected pathways were investigated in more detail within this study.

**Figure S3. Overview of identified key drivers for selected traits.**

**+NASH+MSC vs. +NASH-MSC**


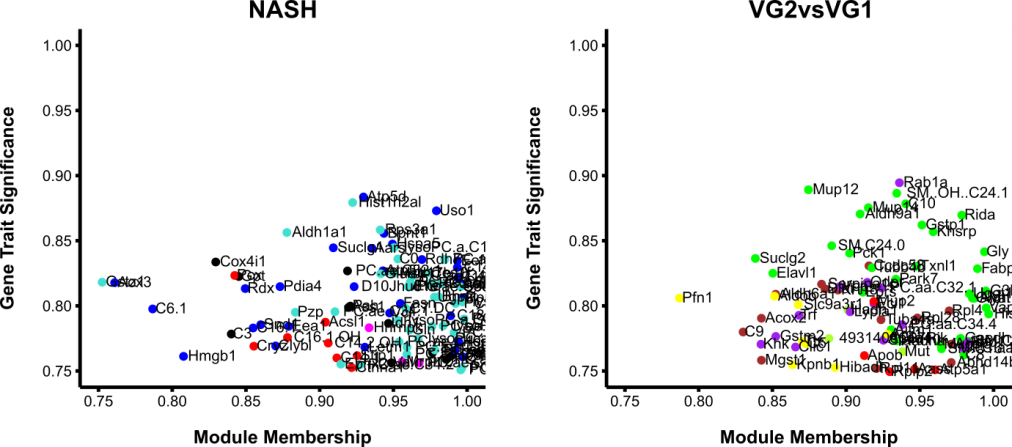


Plotted are analytes that showed absolute gene significance ≥ 0.75 and absolute module membership ≥ 0.75 for the traits that showed highest correlation with at least one module. Analytes are colored based on the module they were assigned to in the WGCNA.

**Figure S4. Biochemical characterization of hepatocyte/MSC cultures.**

**
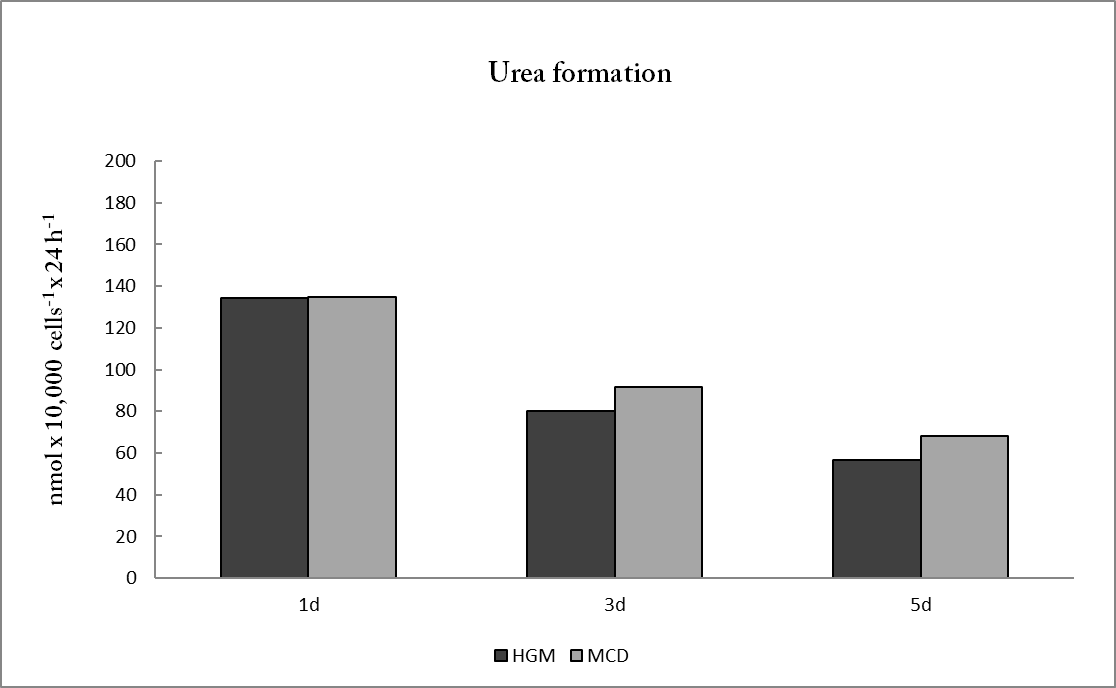
**

**A** Urea synthesis in isolated mouse hepatocytes cultured in HGM or MCD medium for the days indicated. The urea synthesis rate was determined by the diacetyl monoxime method [[1](#_ENREF_1)]. Culture medium was changed 24 h before the measurement. Values are means from two different cell cultures, each run in duplicate. No differences were observed between culture of cells in HGM or MCD medium. The urea synthesis rate decreased by about one half after one day of culture and remained stable until 5 days of culture. This is a general phenomenon with primary hepatocytes as seen in our previous studies with rat and porcine hepatocytes. The functional decrease is not due to the de-differentiation of hepatocytes, but rather due to the lack of the appropriate parenchymal microenvironment of the liver, and may be restored by provision of adequate physiological stimuli [[2](#_ENREF_2)][[3](#_ENREF_3)].


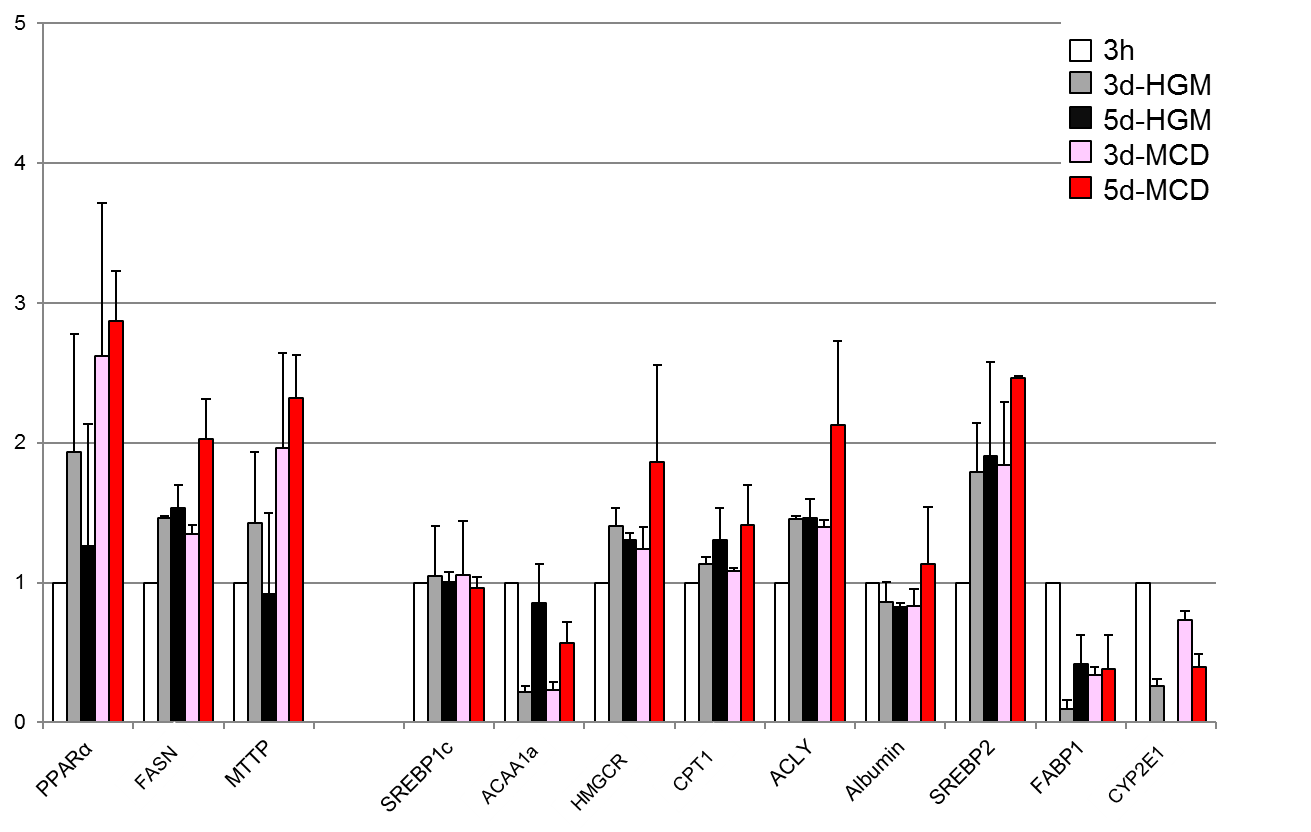


Relative RNA expression (-fold vs. 3 h)

**B** Expression of lipid metabolism genes in isolated mouse hepatocytes cultured in HGM or MCD medium for the days indicated. Gene expression was determined by semiquantitative RT-PCR. Relative expression levels were normalized to levels of β2-microglobulin. Values express x-fold changes vs. expression in hepatocytes after 3 h of culture (representing freshly isolated cells). They are means of two independent cell cultures. Gene expression does not show obvious differences between culture in HGM or MCD medium. Changes over time are similar under all conditions tested. The expression of albumin is equal at each time point and under both culture conditions indicating that the MCD medium may not affect general hepatocyte functions, thus corroborating data shown in (A) for urea synthesis. Abbreviations: ACAA1a - Acetyl-CoA acyltransferase 1; ACLY – ATP citrate lyase; CPT1 – Carnitine palmitoyltransferase 1A; CYP2E1 – Cytochrome P450 subtype 2E1; FABP1 – Fatty acid binding protein 1 (liver); FASN – Fatty acid synthase; HMGCR – 3-Hydroxy-3-methylglutaryl-CoA reductase; MTTP – Microsomal triglyceride transfer protein; PPARα –
Peroxisome proliferator-activated receptor alpha; SREBP – Serum response element binding protein.


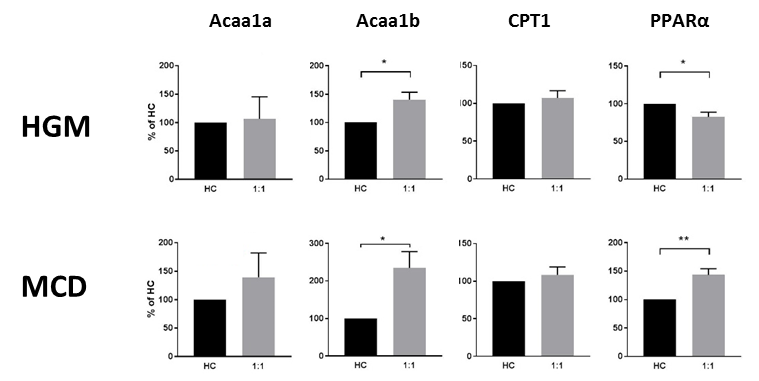


**C** Effects of MSC on the expression of selected lipid metabolism genes after 3 days of co-cultures with hepatocytes in either HGM or MCD medium. Expression was normalized to β2-microglobulin, and levels in hepatocytes set to 100 %, to which the other values are relative. HC indicates hepatocytes alone and 1:1 in co-culture with equal cell numbers of MSC. Values are means from 3 (HGM) and 5 (MCD) different cell cultures, resp. Differences between groups were considered significantly at the level of *p* $\leq$ 0.05 (*) or $p\leq$ 0.01 (**) (Student´s *t*-test for unpaired values).

**Figure S5.** **Formation of tube-like structures between MSC and hepatocytes in co-cultures.**

**
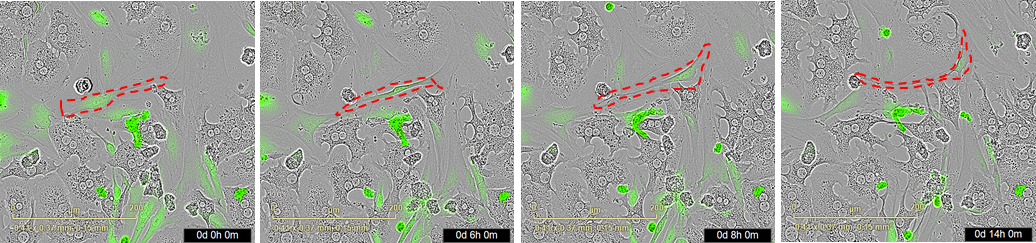
**

The formation of TNT was monitored by live cell imaging using the IncuCyte® S3. After forming a cell-cell contact between the MSC (labeled in green by CFSE (Thermo Fisher Scientific, Germany) and the targeted cell, the MSC moves to the right and is leaving a tubular structure behind (circled by the dotted red line). Cells were co-cultured for 1 day in HGM and pictures were taken every 2 hours. To start the video, open Supplementary file 3. Please pay attention to the cell in the middle of the upper third of the image.

**Figure S6.** **Bi-directional exchange between MSC and hepatocytes in co-cultures via TNT.**

**
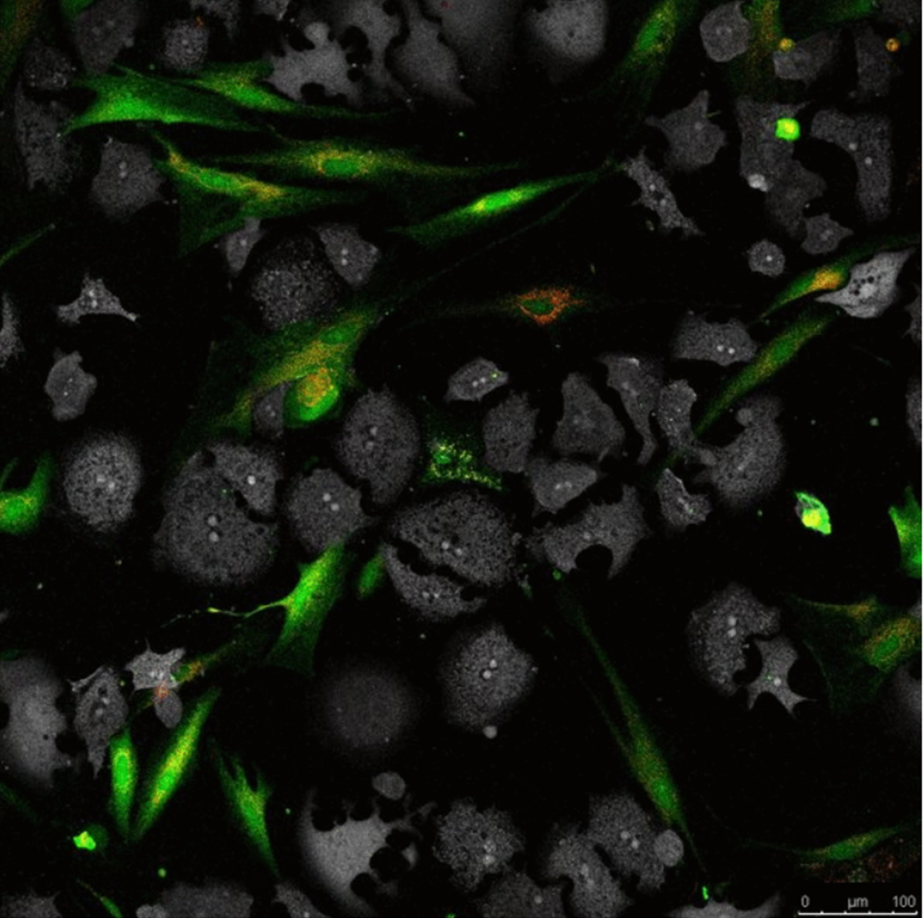
**

**A** Time-lapse confocal imaging of MSC and hepatocyte co-cultures. Human bone marrow-derived MSC were pre-labeled with MitoTracker Deep Red and CellTrace Yellow before co-culture. In the co-culture with hepatocytes, cells were grown in MCD and culture continued for 24 h. Before starting imaging, cultures were stained with CFSE (pseudo-colored white) and pictures taken every 15 min for 23 h and 45 min in total. White and red boxes mark higher magnifications as shown in B and C, resp.


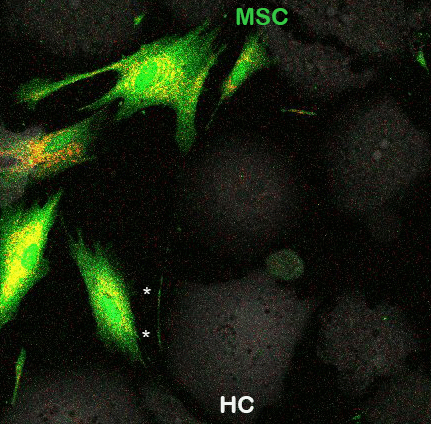


**B** Higher magnification snapshot out of (A, white box) (to see video, please open **Supplementary file 4**). Cells were treated as described in A. MSC present in green and hepatocytes (HC) in pseudo-color white. Human mitochondria present in red label. Stars indicate green MSC-derived material transported to hepatocytes via TNT.


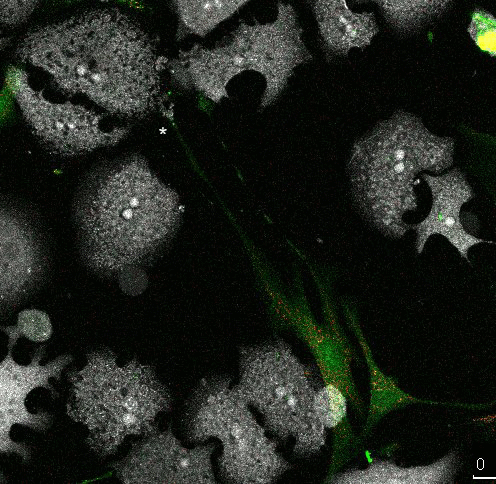


**C** Higher magnification video out of (A, red box) (to see video, please open **Supplementary file 4**). Cells were treated as described in A. MSC present in green and hepatocytes (HC) in pseudo-color white. Human mitochondria present with red labeling. The stars indicate white hepatocyte-derived material transported to MSC via TNT.

**Figure S7.** **Transport of mitochondria and peroxisomes from MSC to hepatocytes via TNT.**

**
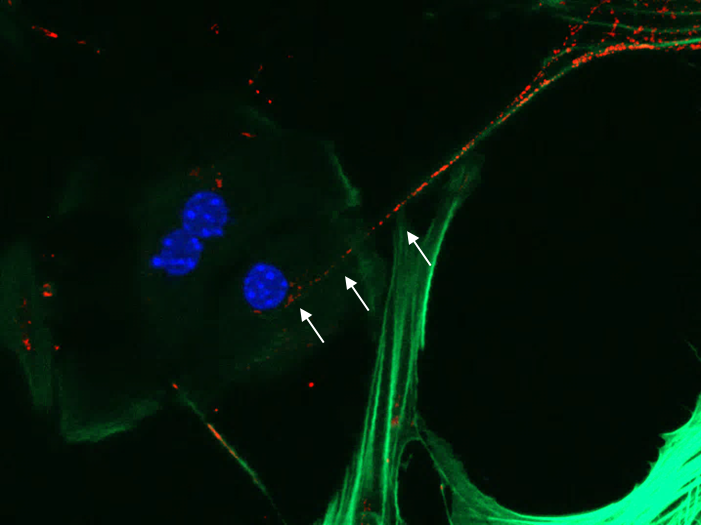
**

**A** Hepatocytes were co-cultured with MitoTracker Red CMXRos (red) pre-labeled MSC. On day 1, cells were further counterstained with Phalloidin-iFluor 405 (pseudo-colored green) to discriminate between mouse hepatocytes and human MSC. MSC-derived mitochondria, labeled in red, are designated by the arrows. Nuclei were stained with DAPI (blue). Pictures were taken by a fluorescence microscope equipped with ApoTome.2. Bar, 100 μm.


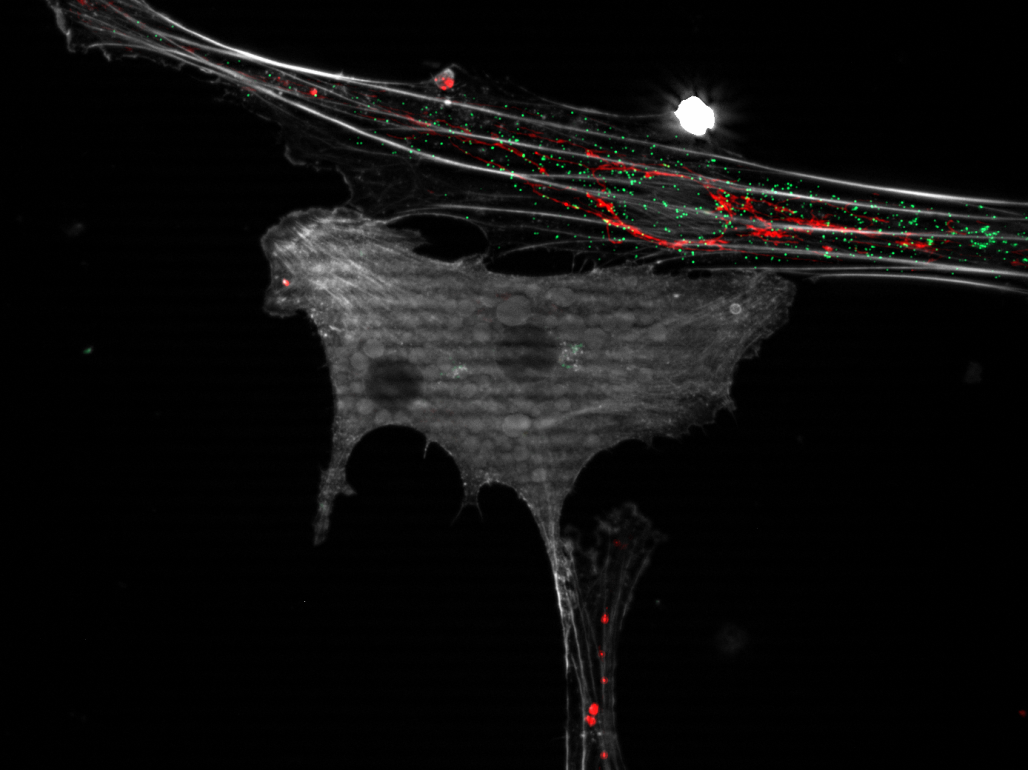


**B** Human MSC-derived peroxisomes, labeled in green with the CellLight™ Peroxisome-GFP, BacMam 2.0 System (Thermo Fisher C10604) are delivered to mouse hepatocytes via TNT. MSC were pre-labeled with MitoTracker Red CMXRos (red) to stain human mitochondria (red), F-actin was stained with Phalloidin-iFluor 405 (pseudo-color white) to discriminate between mouse hepatocytes and human MSC. The solid and dotted arrows point to the MSC-derived MitoTracker Red- and peroxisome-GFP-labeled mitochondria and peroxisomes, respectively, in HC. Pictures were taken by a fluorescence microscope equipped with ApoTome.2. Bar, 100 μm.

*Labeling of peroxisomes using the BacMam system*

One day before the co-culture experiment, hepatocytic differentiated MSC were trypsinized and seeded onto collagen-coated glass coverslips to achieve around 70% confluency. After 3 h, cells were washed twice with PBS and then transduced with CellLight™ Peroxisome-GFP, BacMam 2.0 (Thermo Fisher C10604) at 50 particles per cell in complete cell medium and mixed gently. Cells were ready to split for co-culture experiments the next day.

**References**

1. Crocker, C.L., Rapid determination of urea nitrogen in serum or plasma without deproteinization. *The American journal of medical technology* **1967**, *33*, 361-365.

2. Aurich, H.; Koenig, S.; Schneider, C.; Walldorf, J.; Krause, P.; Fleig, W.E.; Christ, B., Functional characterization of serum-free cultured rat hepatocytes for downstream transplantation applications. *Cell transplantation* **2005**, *14*, 497-506.

3. Schneider, C.; Aurich, H.; Wenkel, R.; Christ, B., Propagation and functional characterization of serum-free cultured porcine hepatocytes for downstream applications. *Cell and tissue research* **2006**, *323*, 433-442.
